# Ambient temperature, suicide, and urbanicity: A nationwide time-stratified case-crossover study in South Korea

**DOI:** 10.1371/journal.pone.0337945

**Published:** 2025-12-16

**Authors:** Harin Min, Jieun Oh, Jiwoo Park, RyangHa Kim, Yejin Kim, Whanhee Lee

**Affiliations:** 1 Graduate School of Data Science, Pusan National University, Busan, South Korea; 2 Department of Public Health Sciences, Graduate School of Public Health, Seoul National University, Seoul, South Korea; 3 Department of Information Convergence Engineering, Pusan National University, Yangsan, South Korea; 4 Graduate School of Data Science, KAIST, Daejeon, South Korea; 5 School of Biomedical Convergence Engineering, College of Information and Biomedical Engineering, Pusan National University, Yangsan, South Korea; 6 The Environmental Center for Climate Change, Pusan National University, Yangsan, South Korea; The University of Tokyo Graduate School of Medicine Faculty of Medicine: Tokyo Daigaku Daigakuin Igakukei Kenkyuka Igakubu, JAPAN

## Abstract

Several studies have examined the nationwide or multi-region relationship between ambient temperature and suicide mortality; however, evidence of comprehensive roles of urbanicity that can affect temperature, suicide, and risk-risk populations was limited. To reduce the gaps in knowledge, this study examined the nationwide ambient temperature-suicide association and heterogeneous high-risk populations by urbanicity levels and sex/age groups, based on a time-stratified case-crossover design with national mortality data (2015–2019). In the total population (65,645 suicides), we found an inverted J-shaped relationship between ambient temperature and suicide, and across all populations, higher temperature was associated with an increased risk of suicide mortality. Nevertheless, the association differed by urbanicity level, sex, and age group. First, in the total population, metropolitan and less-urban areas showed the stronger ORs (odd ratios between the maximum suicide temperature and 20th percentile of the temperature; metropolitan OR: 1.471, 95% CI: 1.141–1.898; less-urban OR: 1.631, 95% CI: 0.968–2.747) than mid-urban areas. In metropolitan areas, stronger ORs were observed in individuals aged 65 years or older (1.926, 1.175–3.157) and 0–44 years (1.575, 0.951–2.608) than in those aged 45–64 years. In mid-urban areas, all subgroups showed no evident association except for people aged 65 years or older. Whereas, less-urban males showed a marginally higher OR (1.667, 0.911–3.051) than less-urban females, although we could not observe sex differences in other areas. Our findings provide evidence for establishing more precise urban health policies and social interventions to reduce the risk of suicide related to ambient temperatures.

## Introduction

Suicide is a crucial global public health concern. The World Health Organization (WHO) report in 2019 reported that approximately 700,000 individuals die due to suicide every year, the numbers are larger than deaths for breast cancer or malaria [1]. Suicide rates in East Asia are relatively higher than in other regions [2], among them, South Korea has experienced several social problems related to the higher suicide rates. OECD reported that the suicide rate was the highest (24.1 suicides per 100,000 persons) among the OECD countries in 2020, which was more than twice the OECD average (11 suicides per 100,000 persons) [3]. Especially, in South Korea, related to rapid aging and resultant aftereffects, the suicide rate of senior populations (individuals aged 65 years or older) was 47.7 suicides per 100,000 people in 2017: it was approximately twice as high as the suicide rate of individuals aged 15–64 (24.3 suicides per 100,000 people) and also greater than the OECD average (18.8 suicides per 100,000 people) [4].

The risk factors of suicide behavior are complex, with multiple reasons that can predispose people to attempt to take their lives [5,6]. Previous literature demonstrated that economic, sociological, psychological, and genetic or biological factors are related to suicidal behaviors [7–9]. In addition, environmental factors, particularly ambient temperatures, have been revealed as one of the major risk factors for suicide [6,10]. A recent study reported that excess suicide mortality attributable to ambient temperatures was around 4.2%, and it is expected to increase up to a maximum of 6.5% in 2090–2099 [11]. Increases in discomfort moods, impulsive-aggressive behaviors, and frustration by exposures to high temperatures suggested as putative biological mechanisms [8,12], and an existing study also reported the association between ambient temperature and decreased biomarkers of serotonin [13], which are closely related to mental disorders, such as anxiety and panic [14]. Therefore, finding complex epidemiological roles of ambient temperatures on suicide mortality can provide informative evidence for improving related public health plans and preemptive interventions.

Urbanicity, which indicates the impact of living in urban areas [15], is also a complex concept related to lifestyles, ecosystems, social disparities, as well as climate change, and mental health [16–18]. Previous studies found that higher mortality risks related to hot temperatures were observed in metropolitan areas compared to peri-urban or rural areas, and they suggested this pattern might be related to urban heat islands, less vegetation, high social isolation, and poor accessibility to medical infrastructure due to a large number of resident people [16,19–21]. Further, earlier studies revealed that urbanicity-related characteristics, such as social connectedness, access to social infrastructures or leisure services, and socioeconomic status, were associated with a suicide rate [18,22,23]. Despite the comprehensive and important roles of urbanicity related to both suicide and temperatures, few studies examined this issue [24].

To address the knowledge gaps, this study evaluated the nationwide association between ambient temperature and suicide mortality, particularly related to the complex roles of urbanicity. The study examined heterogeneous temperature-suicide mortality relationships depending on urbanicity, age, and sex to find high-risk populations regarding suicide risk due to ambient temperatures using the national mortality data from 2015–2019 in South Korea.

## Materials and methods

### Ethical approval

Not requested. This study used secondary and publicly available data. This data did not include any information related to the personal identification.

### Study population and suicide data

The study population of this study includes all individuals who died due to suicide and were registered in Statistics Korea. Specifically, we obtained individual-level mortality data in the entire 247 districts in inland South Korea (except Jeju and Ulleung islands because of the absence of exposure data) from 1^st^ January 2015–31^st^ December 2019. The mortality data is completely de-identified and publicly available. The International Classification of Disease 10^th^ Revision (ICD-10) was used to define suicide mortality (ICD-10: X60–X84) [2]. Potential errors may occur when deaths are underreported or when causes of death are misclassified. To address these limitations, Statistics Korea integrates additional data sources— such as the cause of death supplementary survey, infant cremation records, and data on unclaimed deceased persons —to improve the accuracy of mortality statistics.

### Urbanicity indicator

We selected the population density (persons per km^2^) to approximate the urbanicity level of each district. It was suggested as one of the most suitable indicators that can reflect the urbanicity level in South Korea in a previous study [16]. Although we recognized that metrics for the determination of the urbanicity level and the thresholds of urban and rural areas were highly heterogeneous by regions and countries, if the point that Korea is one of the most densely populated countries among those in the OECD was considered [25], population density may be the most appropriate urbanicity indicator in South Korea. Further, around 45% of the population lives in the seven largest metropolitan cities which account for only 5% of the total area, and highly urbanized districts in South Korea (especially in Seoul and Busan metropolitan cities; the two largest cities in Korea) show the highest population densities [16]. Therefore, this study selected population density as an indicator of urbanicity level for each district. Then, for each district, we averaged annual population density across the study period and categorized districts into three categories based on the tertiles [26] of the population density averages: Metropolitan areas (above 66.7^th^ percentile), mid-urban areas (between 33.3^rd^ and 66.7^th^ percentiles), and less-urban areas (below 33.3^rd^ percentile).

Furthermore, recently in South Korea, economic problems (25.0%) and physical diseases (20.6%) were primary and secondary motivations of suicide, except for mental disorder problems (31.7%) [4]. Urbanicity was closely related to socioeconomic status and medical services [16], thus we also collected three related district-level indicators that can be surrogate variables to imply levels of socioeconomic status and medical services related to urbanicity: 1) the % of people who receive the National Basic Livelihood Security Service (% Basic livelihood security recipient), 2) the % of people who could not visit the medical facilities when they wanted within a year (% Unmet medical needs), and 3) the % of people who could not visit the medical facilities when they wanted within a year because of economic reasons (% Unmet medical needs due to economic reasons). Like population density, we averaged all these three indicators across the study period and categorized them into three categories (Low, Middle, and High) based on their tertiles. These indicators were obtained from the national community health-related database [27].

### Environmental data

As a main exposure, we collected daily reanalyzed 2-m ambient temperatures (°C) from the Google Earth Engine with the ERA-5 Land dataset for 2015–2019 [28]. The satellite-based daily temperature data were extracted in a 0.1° spatial grid, thus we aggregated it into district units (known as “*si/gun/gu*” in Korean) [29]. The reanalyzed temperatures showed a high consistency (R^2^ > 0.96) with real-time monitored temperatures by the Korean Meteorological Office (KMO). Because the monitoring stations of KMO were placed sparsely (on average, one or two stations are in each district, and some districts had no station), we used the satellite-based daily temperatures as a main exposure variable. We also collected dewpoint temperature (K) from the ERA-5 Land dataset.

We also obtained daily district-level modeled fine particulate matter (24-hour average PM_2.5_; μg/m^3^) and ozone (maximum 8-h average O_3_; ppm) from the AiMS-CREAT team. The team developed machine-learning ensemble prediction models for PM_2.5_ and O_3_ with excellent prediction accuracy (validated R^2^ values: 0.944 for PM_2.5_ and 0.944 for O_3_) and high resolution (1 km^2^), and these ensemble models were used in published studies [30,31]. These models covered all 247 districts in inland South Korea. More detailed information on the air pollution prediction models is provided in the Supplementary Materials (S1 File), “1. Air pollution prediction models”. Table S1 in S1 File shows the performance of the prediction models.

### Subgroup analysis

In addition to three-categorized areas by urbanicity level, we examined age groups and sexes as subgroups to find more detailed high-risk populations. Age and sex have been recognized as potential effect modifiers, however, the corresponding results were heterogeneous by studies [6,32–34]. We divided ages at death into four categories: total (all ages), individuals aged 0–44 years, 45–64 years, and 65 years or older. We estimated the association between temperature and suicide mortality by sex and age group, individually, and the sex and age categories were also used for stratified analyses by urbanicity levels (metropolitan/mid-urban/less-urban areas).

Further, to partly explain the differences depending on urbanicity level, we analyzed three district-level socioeconomic indicators associated with urbanicity. First, we did an ANOVA (analysis of variance) to examine whether the three indicators are related to levels of urbanicity. Then, we did a stratified analysis for the total population to evaluate whether the relationship between ambient temperature and suicide death is modified by the level of each urbanicity-related indicator (low/middle/high).

### Statistical analysis

As the main analysis, we created a time-stratified case-crossover dataset for each combination consisting of three urbanicity levels, three age groups, and sexes (a total of 18 combinations were used: three urbanicity levels and three age categories, and two sexes). We identified a case day as the date of suicide death and matched control days as days with the day of the week within the same month in the same year. This self-matching by the same month and year can control for confounding variables that do not markedly change within a month, such as age, sex, diet, body mass index, and regional indicators (accessibility to social facilities, demographic compositions, regional socioeconomic status, climate, etc.). Bidirectional matching by day of the week within the same month and year controlled potential suicide mortality differences within a week, seasonality, and long-term trends [31]. This approach has been widely used to assess the association between short-term exposure to environmental stressors and health outcomes [29,35,36].

For each case-crossover dataset, we performed a conditional logistic regression with a distributed lag nonlinear model to estimate the association between ambient temperatures and suicide mortality. First, to consider the heterogeneous temperature distributions across districts, we transformed the absolute temperature to relative temperature (the % of temperature) for each district. This procedure allows all districts to have the same ranges of ambient temperatures (0–100%), and the use of the percentile scale temperatures is a standardized approach in a multi-region study on temperature and health to reflect different distributions of temperatures for each region [6]. The relative temperature metric is computed based on the empirical cumulative distribution function (simply using the *“ecdf”* package in R). Then, we modeled the association between ambient temperatures and suicide mortality with a distributed lag nonlinear model function: for the exposure-response relationship, we used a natural cubic spline with three internal knots placed at the 25^th^, 50^th^, and 75^th^ percentiles of district-specific temperature distributions. For lag-response association, we used three-day lag periods for temperature (lag 0–2) and applied a natural cubic spline with an internal knot at lag day 1. The model specifications regarding the exposure-lag-relationships between ambient temperature and suicide mortality were based on previous large data studies [6,32] as well as our grid search to find an optimal number and spaces of knots and lag days based on the Akaike Information Criteria (with a partial likelihood). The daily modeled O_3_ (lag 0–2), PM_2.5_ (lag 0–2), dewpoint temperature (lag 0–2), and indicators of the holiday were adjusted in all conditional logistic regression models. For each model, we reduced the parameters from the distributed lag nonlinear model to estimate the lag-cumulative temperature-suicide association [37].

From the estimated lag-cumulative dose-response curves, we calculated odd ratios (ORs) between a reference temperature percentile (20%; around 2.18 °C) and the maximum suicide temperature (MaxST). We selected it as a primary OR calculation procedure because previous studies exhibited that the temperature-suicide association in East Asian regions had an inverted J-shaped curve in general (i.e., a higher temperature was related to an increased risk of suicide, but the risk decreased at extremely high temperatures at around 90–99^th^ percentiles) [6,11]. As a secondary risk measurement, we calculated ORs for an increase in 10 °C of ambient temperature using a conditional logistic regression with a linear term of temperature metric (lag 0–2), instead of the spline function.

Additionally, to examine the effect modifications by three urbanicity-related indicators in the total population, we created nine more case-crossover datasets (three indicators classified into three categories) and repeated the main analysis for each dataset to estimate the different associations between temperature and suicide by levels of each indicator.

Lastly, we performed sensitivity analyses to examine whether our results are consistent with different modeling specifications. As a supplementary analysis, absolute metrics were examined to verify that the observed trends were consistent with those based on relative measures. The results are provided in the Results section. In all statistical analyses, R software (version 4.2.1) with libraries “*dlnm*” and “*survival*” were used.

## Results

Table 1 presents the descriptive information on suicide deaths during the study period (2015–2019). This study included a total of 65,645 suicide deaths, and suicides per 100,000 persons (i.e., baseline risk) for five years was 126.88 (25.3/year) in the total population. Metropolitan areas showed the largest suicide deaths (50.90%), but the baseline risk was the highest in less-urban areas. Males and individuals aged 65 years or older showed the larger numbers of suicide deaths (46,649 suicides, 17,797 suicides) and baseline risks (180.57 suicides per 100,000 people, 241.78 suicides per 100,000 people), compared to females and other age groups, respectively.

**Table 1:** Descriptive information on suicide mortality data during the study period (2015–2019).

	Number of Suicides	(%)	Suicides per 100,000 persons*	Temperature(°C)	
				Mean	Standard Deviation
**Total**	65,645	100.0	126.88	12.90	1.32
**Metropolitan areas**	33,411	50.90	120.16	12.89	1.08
**Mid-Urban areas**	25,026	38.12	134.24	13.01	1.12
**Less-Urban areas**	7,208	10.98	164.88	12.81	1.69
**Male**	46,649	71.06	180.57		
**Female**	18,996	28.94	73.34		
**0-44 years**	22,242	33.89	78.32		
**45-64 years**	25,595	38.99	160.19		
**65 year or older**	17,797	27.11	241.78		

*Average population during the study period was used.

**Fig 1:**
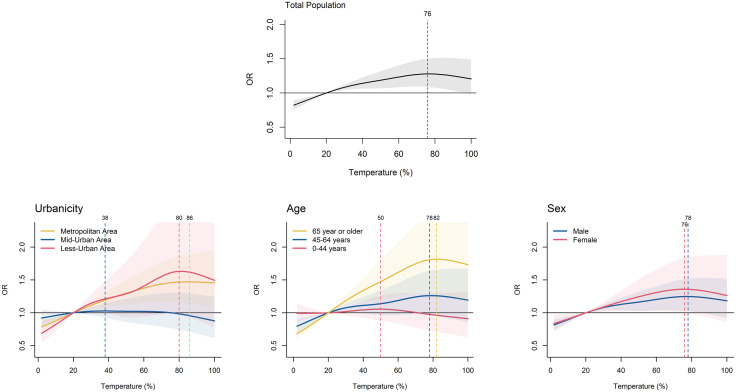
Lag-cumulative associations between ambient temperature and suicide in the total population and by subgroup. OR: Odd ratio (a reference point: 20^th^ percentile of the temperature distribution). Dashed lines indicate the maximum suicide risk temperature percentiles (MaxSTs).

Fig 1 exhibits the lag-cumulative ambient temperature-suicide association in the total population and by subgroups (urbanicity categories, age groups, and sexes). Hereafter, all the associations between temperature and suicide indicate the lag-cumulative association. In the total population, the association showed the inverted J-shape and the MaxST was at the 76^th^ percentile of the temperature distribution (~21.1 °C). The association curves were heterogeneous by subgroup, although the statistical evidence regarding the differences was generally weak. First, among the three categories of urbanicity level, the association between temperature and suicide mortality was the highest in metropolitan and less-urban areas (MaxST: 80^th^, 86^th^ percentile), and it was the lowest in mid-urban areas (MaxST: 38^th^ percentile). Second, individuals aged 65 years or older showed the highest association between temperature and suicide mortality (MaxST: 82%), compared to other younger age groups. Third, females showed a slightly higher association with temperatures, but the difference was not prominent. MaxSTs of male and female were 76%, 78%, respectively. Table S2 in S1 File shows the distributions of absolute temperatures with their corresponding percentiles. Absolute metrics further confirmed the trends observed with relative metrics and are presented for reference only (Fig S1 in S1 File).

**Fig 2:**
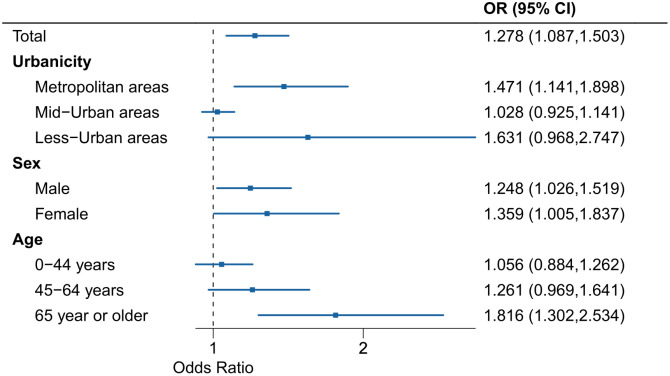
Lag-cumulative ORs between a reference temperature percentile (20^th^ percentile) and MaxST in the total population and by urbanicity level and sex/age group. OR: Odds ratio. MaxST: Maximum suicide temperature percentile.

Fig 2 shows the (lag-cumulative) ORs between a reference temperature percentile (20^th^ percentile) and MaxST in the total population or subgroups. In the total population, the estimated OR was 1.278 with 95% CI: 1.087, 1.503. Metropolitan (OR: 1.471, 95% CI: 1.141, 1.898) and less-urban(OR: 1.631, 95% CI: 0.968, 2.747) areas showed the higher OR compared to the mid-urban area, and the difference between metropolitan and mid-urban areas was statistically evident (no overlap between CIs). We could not observe a pronounced difference between the sexes. Whereas regarding age groups, OR estimates tended to increase as age increased, and OR for individuals aged 65 years or older was 1.816 (1.302, 2.534). Fig S2 in S1 File showed OR estimates corresponding to Fig 2, based on the assumption of a linear association between ambient temperature and suicide mortality risk. The risk patterns by subgroups were consistent even when the linear association was applied.

**Fig 3:**
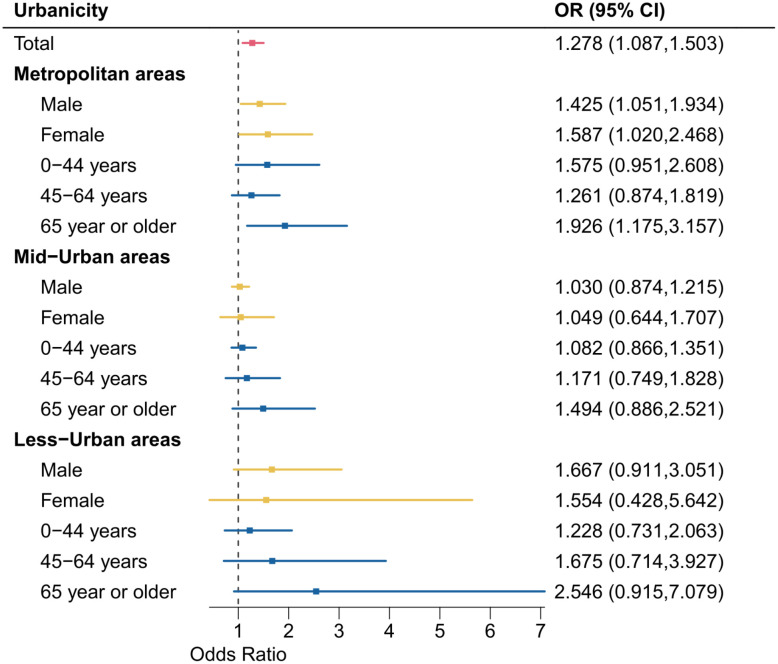
Lag-cumulative ORs between a reference temperature percentile (20^th^ percentile) and MaxST by urbanicity level and sex/age groups. OR: Odds ratio. MaxST: Maximum suicide temperature percentile.

The results of double-stratification analyses by urbanicity level and sex/age group are presented in Fig 3. We observed a pattern that, based on the point estimates, individuals aged 65 years or older showed the highest ORs compared to other younger age groups across all areas divided by urbanicity. While, in metropolitan areas, people aged 0–44 years showed a stronger OR (1.575 with 95% CI: 0.951, 2.608) estimate than those aged 45–64 years. In all areas, although differences in estimated ORs by sex were statistically weak, males showed a higher OR than females only in less-urban areas. Fig S3 in S1 File showed OR estimates corresponding to Fig 3 based on the linearity assumption, and the estimated patterns in Figs 3 and S2 in S1 File were generally consistent.

**Fig 4:**
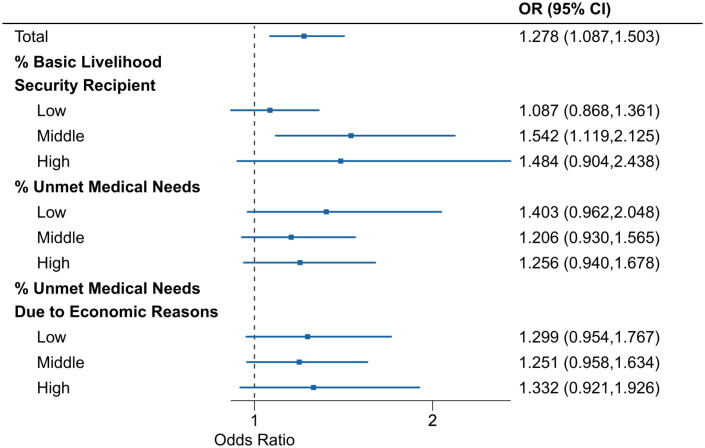
Lag-cumulative ORs between a reference temperature percentile (20^th^ percentile) and MaxST by urbanicity-related indicators. OR: Odds ratio. MaxST: Maximum suicide temperature percentile. The % of people who receive the National Basic Livelihood Security Service (% Basic livelihood security recipient), the % of people who could not visit the medical facilities when they wanted within a year (% Unmet medical needs), and the % of people who could not visit the medical facilities when they wanted within a year because of economic reasons (% Unmet medical needs due to economic reasons).

Table 2 showed ANOVA results that all three indicators were related to the urbanicity level. % Basic Livelihood Security Recipient and % Unmet Medical Needs were the lowest in less-urban areas than other areas, however, % Unmet Medical Needs Due to Economic Reasons was the highest in metropolitan areas. Fig 4 exhibits the effect modifications by urbanicity-related indicators that might cover regional socioeconomic and medical service levels. Further, although the statistical evidence of the effect modifications was insufficient, the effect modification by % Basic Livelihood Security Recipient showed a slightly more prominent modification compared to the other two indicators, and the category of the moderate % Basic livelihood security recipient showed the highest OR estimate compared to all other subcategories regarding urbanicity-related indicators. Fig S4 in S1 File showed OR estimates corresponding to Fig 4 based on the linearity assumption.

**Table 2:** Analysis of variance (ANOVA) results that all three indicators were related to the urbanicity level.

	Metropolitan areas		Mid-Urban areas		Less-Urban areas		P-value
	Mean	Standard Deviation	Mean	Standard Deviation	Mean	Standard Deviation	
% Basic Livelihood Security Recipient	33.03	13.72	31.65	13.00	49.51	10.36	<0.001
% Unmet Medical Needs	6.08	2.38	6.78	3.54	7.01	4.06	<0.001
% Unmet Medical Needs Due to Economic Reasons	0.72	0.58	0.61	0.43	0.47	0.40	<0.001

The % of people who receive the National Basic Livelihood Security Service (% Basic livelihood security recipient), the % of people who could not visit the medical facilities when they wanted within a year (% Unmet medical needs), and the % of people who could not visit the medical facilities when they wanted within a year because of economic reasons (% Unmet medical needs due to economic reasons).

Lastly, sensitivity analyses show that our main results in the total population and by urbanicity remained robust to different modeling specifications (Table S3 in S1 File). Fig S5 in S1 File shows that the results remain robust across sensitivity analyses with varying lag assumptions, which is consistent with the results reported in Table S3 in S1 File. Further, we observed that the size of OR estimates without adjusting dewpoint temperatures decreased.

## Discussion

In this study, we assessed the nationwide association between ambient temperature and suicide in South Korea with national mortality and meteorological data. In particular, this study has a novelty in addressing the heterogeneous temperature-suicide mortality relationships and different high-risk populations by urbanicity level. Metropolitan and less-urban areas exhibited the higher association between temperature and suicide than mid-urban areas. We also found that individuals aged 65 years or older showed the highest association between temperature and suicide across all areas, whereas relatively young individuals (aged 0–44 years) showed a noticeable relationship between temperature and suicide only in metropolitan areas. Metropolitan and mid-urban areas had no obvious differences by sexes; however, in less-urban areas, males showed a marginally higher risk of temperature to suicide than females.

Our study results in the total population or in metropolitan areas were generally consistent with previous findings based on monitored metropolitan or urban areas. A recent nationwide study in Japan revealed that the association between ambient temperature and suicide mortality has an inverted J-shaped exposure-response curve [11]. A multi-country study in 341 locations (mostly urban cities) in 12 countries found that the inverted J-shaped association between ambient temperature and suicide mortality was observed in northeast Asia, while European countries and North American countries showed an approximately linear relationship between ambient temperature and suicide. This study suggested that different climates are plausible explanations for the difference (a linear association observed in European and North American countries might be due to fewer hot days in their climates. It is related to a narrower range of temperature distribution that can show some linear parts of the overall inverted J-shaped temperature-suicide association) [6]. A nationwide study in France supported this hypothesis by evaluating approximately linear relationships between temperature and suicide across 50 years [10].

Our results show an inverted-J shape, which is consistent with previous studies. While the scale of the estimated risks varies—likely due to differences in exposure metrics, methods of calculating OR/RRs, or the study region and period—the overall pattern of the results remained comparable. In the Japanese study [11], MaxST was found to be 23.1 °C. This result was obtained by performing absolute temperature-based modelling for each Japanese prefecture and then applying multivariate meta-regression. In the multi-country study [6], mentioned earlier, percentile temperature was used, which is the same relative temperature metric as in our study. Modelling for six regions in Korea revealed that MaxST was obtained at 88%, or approximately 25 °C. In our study, MaxST was observed at 76%, and this value was found to be 21.13 °C in the Table S2 in S1 File. In the former, RR was estimated based on the 25^th^ percentile (7.7 °C), showing a directionality of approximately 4% increase in RR. In the latter, RR estimated based on MaxST compared to MinST is presented as 1.61. In our study, when calculating the OR, we used the 20^th^ percentile (2.18 °C) as the reference point for MaxST, resulting in an OR of approximately 1.28. Additionally, in Fig S2 in S1 File—linear OR, which shows the OR per 10 °C increase—although not statistically significant, there is a trend indicating that a 10 °C increase in absolute temperature is associated with approximately a 1% increase in risk.

Interestingly, to our knowledge, although several studies addressed the nationwide association between temperature and suicide mortality [10,11,38,39], few studies have examined the roles of urbanicity or urban-rural differences in the temperature-suicide association [40]. We found only one epidemiological study in Japan evaluated the urban-rural difference in the association and examined the plausible factors (lower education level, higher percentages of seniors and unemployment, and lower air conditioner prevalence) that might be related to the difference [40]. The study design employed a two-stage approach, incorporating a time-stratified case-crossover design and multivariate meta-regression, which examined an elevated risk among female, elderly, and rural populations. However, this study did not address heterogeneous high-risk populations by urbanicity.

The major novel finding of this study is that this study revealed different high-risk populations regarding the temperature and suicide association by regional urbanicity. First, unlike a previous study in Japan [40], we found that people residing in metropolitan areas or less-urban areas marginally showed a stronger temperature-suicide association compared to mid-urban areas. Second, high-risk populations differed by urbanicity. We found that individuals aged 65 years or older showed a higher risk compared to other age groups across all areas classified by urbanicity level, however, individuals aged 65 years or older residing in metropolitan areas had a higher temperature-suicide association than those living in mid- or less-urban areas. Further, unlike mid- or less-urban areas, people aged 0–44 years had a slightly evident temperature risk estimate of suicide, and it was higher than people aged 45–64 years in metropolitan areas.

There are two plausible explanations for these findings. First is the heat island phenomenon in metropolitan areas. Especially, districts in or neighboring Seoul Busan metropolitan cities (mostly included in metropolitan areas) have shown the obvious urban heat anomaly [19] and the urban heat island phenomenon has been increasing over time [41]. Numerous studies have identified that the increases in temperatures might lead to aggressive thoughts, feelings, and behaviors [34,42,43], and a recent study found that nighttime temperatures, especially related to the urban heat anomaly, were associated with an increased risk of suicide [44]. Thus, greater exposure to hot temperatures in metropolitan areas can lead to a higher association between suicide and temperature relationship, compared to other areas. Moreover, we conjecture that the urban heat island and resultant higher nocturnal temperatures could affect a high temperature-suicide association in young populations. The percentage of the economically active population has been the highest in metropolitan areas in South Korea, especially among young adults, and most daytime works are indoor works [27]. Thus, people in metropolitan areas likely play outdoor activities during the nighttime after working, which indicates that the economically active young population might be more vulnerable to exposure to hot nighttime temperatures. It should be noted, however, that the daily average temperature used in the study is not suitable for addressing hourly temperature changes. However, the daily average temperature is an aggregate of hourly temperatures and can serve as a partial indicator of phenomena such as the heat island effect and tropical nights. In addition, as the mortality data utilised in this study is aggregated on a daily basis, it would be more appropriate to employ daily temperature indicators as compared to hourly indicators. Therefore, in this study, daily temperature data was used as the exposure variable, enhancing the comparability with previous studies [6,32,40].

Second are the socioeconomic and medical accessibility-related explanations. As we shown in Table 2, metropolitan areas had a higher % Basic livelihood security recipients than mid-urban areas and the highest % Unmet medical needs due to economic reasons compared to other areas. These statistics imply that the proportion of socially marginalized populations might not be low at least in metropolitan areas when considering their large number (~55%) [27], and the absolute number of socially marginalized people might be larger in metropolitan areas than in other areas. Further, a previous study in South Korea revealed that higher urbanicity was associated with lower levels of social trust and connectedness, and higher social isolation [21], which are well-known risk factors for suicide. In addition, due to the extremely high population concentration in metropolitan areas, accessibility to general and emergency medical facilities has been lower than in peri- or mid-urban areas in South Korea [16]. Previous studies in South Korea reported that this limited accessibility to medical services was related to a higher risk of mortality related to environmental stressors [16,45], and we surmised that especially the low accessibility to medical services in metropolitan areas could be linked to higher death risk after suicide attempts.

Another major novel finding of this study is that sex differences in the association between temperature and suicide risk might change in less-urban or rural areas. The differences in the temperature-suicide relationship by sex have been heterogeneous by study regions [6]. Previous studies consistently have reported that the temperature-suicide association was higher in females than in males at least in northeast Asian regions, such as Japan [40], Taiwan [6], Korea [6], and some large cities, such as Beijing, Chengdu, and Guangzhou in China [33]. Our results regarding the total population also showed a higher temperature-suicide association in females, although the statistical evidence was weak. However, we observed that the sex difference might be reversed in less-urban areas. We cautiously surmise that this result might be linked to unique rural socioeconomic activities in South Korea. It has been reported that the traditional social norm that “Men should work in the labour market and women should work at home” is still prevalent throughout Korean society [46]. Differences in economic activity between the sexes are more pronounced in rural areas. In rural areas, a large proportion of the economically active population works outdoors during the day, resulting in more prolonged and intense exposure to extreme temperatures compared with other regions [47]. Furthermore, gender disparities in the type of labour are evident. In rural areas of Korea, men are more likely to operate large tools or machinery and carry heavy objects outdoors, whereas women are more likely to perform indoor tasks or manual work [48]. This indicates that men are more frequently engaged in strenuous physical labour and experience longer exposure to environmental factors in their workplaces. There are also differences in social activity depending on gender in rural areas. It has been reported that women tend to have larger social networks than men [49], while women’s social support derives from a variety of sources, whereas men often rely primarily on emotional fulfilment from their spouses [50]. Further evidence of social isolation among elderly men in rural settings has also been documented [51]. Such disparities in social activity may contribute to differences in loneliness, depression, and suicidal ideation. Consequently, rural elderly men, who are more prone to loneliness, may be more vulnerable to suicide than women due to their socioeconomic circumstances. However, because quantified evidence supporting this hypothesis is limited, this hypothesis should be examined in-depth in future studies with more target-concentrated cohorts.

Several limitations should be discussed. First, we used district-average ambient temperatures as a surrogate of individual temperature exposures because the national mortality data we used provided only district-level addresses (the median size of districts in South Korea is 397 km^2^, which is approximately 1.7 times larger than the median size of the US Zip code areas). Thus, potential exposure misclassification errors could exist. Second, although we adopted a time-stratified case-crossover design to control for confounders that did not substantially change within a month, unmeasured time-dependent confounders, such as daily mood, medication, or high-risk drinking, might affect our risk estimates. Thus, our results are limited in being interpreted as a causal association between temperature and suicide. Third, due to the limited sample size of each stratum, we could not perform an urbanicity-age-sex stratification analysis that could provide more specific information on high-risk populations. Further studies with longer data periods should consider this analytic strategy to construct more targeted action plans and social interventions against suicide and climate change.

## Conclusions

In summary, to our knowledge, this is the first and largest study evaluating the nationwide association between ambient temperature and suicide mortality in South Korea, together with heterogeneous high-risk populations related to the complex roles of urbanicity. People living in metropolitan areas, especially metropolitan young adults (0–44 years) and seniors (65 years or older), had a higher association between temperature and suicide compared to other subpopulations residing in areas with less urbanicity. Further, males in less-urban areas showed a higher association than less-urban females, although sex differences were not observed in metropolitan or mid-urban areas. Our findings provide epidemiological evidence for more precise urban health policies and interventions to reduce the hazardous impacts of temperature on suicide.

## Supporting information

S1 File Supplementary materials. (DOCX)
