# Supplementary material for: Ambient temperature, suicide, and urbanicity: A nationwide time-stratified case-crossover study in South Korea
Source: PLoS One. 2025 Dec 16;20(12):e0337945. doi: 10.1371/journal.pone.0337945 (PMC12707675; doi:10.1371/journal.pone.0337945)
Supplement: S1 File — (DOCX) [file pone.0337945.s001.docx]

**Supporting Information**

**Title:** **Ambient Temperature, Suicide, and Urbanicity: A Nationwide Time-Stratified Cases-Crossover Study in South Korea**

Harin Min^1^, Jieun Oh^2^, Jiwoo Park^3^, RyangHa Kim^4^, Yejin Kim^3^, and Whanhee Lee^5, 6¥^

**Affiliations**

1 Graduate School of Data Science, Pusan National University, Busan, South Korea.

2 Department of Public Health Sciences, Graduate School of Public health, Seoul National University, Seoul, South Korea.

3 Department of Information Convergence Engineering, Pusan National University, Yangsan, South Korea.

4 Graduate School of Data Science, KAIST, Daejeon, South Korea

5 School of Biomedical Convergence Engineering, College of Information and Biomedical Engineering, Pusan National University, Yangsan, South Korea.

6 The Environmental Center for Climate Change, Pusan National University, Yangsan, South Korea.

**Corresponding Author: Whanhee Lee**

School of Biomedical Convergence Engineering, College of Information and Biomedical Engineering, Pusan National University, 49 Busandaehak-ro, Mulgeum-eup, Yangsan-si, Gyeongsangnam-do 50612, South Korea. Telephone: (82) 51-510-8599. E-mail: whanhee.lee@pusan.ac.kr

**1. Air Pollution Prediction Model**

This model was provided air pollution data by AiMS-CREATE team (hereafter “the team”), which is a research network for environmental health between Pusan National University and Seoul National University in Korea, and their products were used in previously published studies.^1,2^

**(1) Information on monitoring station**

As response variables for the air pollution prediction modeling (i.e. as true values), we collected ground-level hourly measured PM_2.5_ and ozone concentrations from the Air Korea database provided by the Ministry of Environment (URL: <https://www.airkorea.or.kr/>) Korea from Jan. 01, 2015 to Dec 31, 2022. To reduce potential biases, we used concentration data from monitoring sites with observations for ≥ 9 months per year (observed 75% or over). The total number of selected monitoring sites was 483 for PM_2.5_ and ozone (105 stations in 2015 to 483 stations in 2022). From the selected monitoring sites, we calculated the average ambient concentrations of daily PM_2.5_ and ozone.

**(2) Explanatory variables for prediction models with single machine learning algorithms**

**(2.1) Satellite-derived data**

Daily variables based on satellite remote sensing from 2015 through 2022 were used as major predictors. First, the team used 1 km^2^ grid cell shape file provided by Statistics Korea. Then, the team collected a total of 47 daily remote sensing variables through the Google Earth Engine (URL: <https://earthengine.google.com/>), and these variables included daily aerosol optical depth, meteorological, surface reflectance and landcover data from different satellite-based databases (the Table below). If the spatial resolution of certain variables was higher than 1km^2^, then we calculated and allocated the average values for each variable inside the boundary of each 1 km^2^ grid cell. Also, if the spatial resolution of certain variables was lower than 1 km^2^, we allocated the nearest values to the centroid of the 1 km^2^ grid cells and calculated the average values when two or more values were included in each 1 km^2^ grid cell Then, we used the *missForest* method to impute missing values in 1 km^2^ grid cell which were not measured with each satellite. Finally, we standardized the satellite-driven data to make zero-mean and unit-variance.

**Table. Information on the explanatory variables from the satellite-based databases in this study for ozone and PM_2.5_ prediction models with single machine learning algorithms**

| **Data source** | **Predictor variables** | **Spatiotemporal**  **Resolution** |
| --- | --- | --- |
| ERA5-Land Daily Aggregated - ECMWF Climate Reanalysis | Temperature_2m | 11.13 km, Hourly |
|  | Skin_temperature |  |
|  | Soil_temperature_level_1 |  |
|  | Leaf_area_index_low_vegetation |  |
|  | Leaf_area_index_high_vegetation |  |
|  | Total_precipitation |  |
|  | Surface_pressure |  |
|  | u_component_of_wind_10m |  |
|  | v_component_of_wind_10m |  |
| MOD09GA.061 Terra Surface Reflectance Daily Global 1km and 500m | Sur_refl_b01 | 500 m, Daily |
|  | Sur_refl_b02 |  |
|  | Sur_refl_b03 |  |
|  | Sur_refl_b07 |  |
| MOD11A1.061 Terra Land Surface Temperature and Emissivity Daily Global 1km | Emis_31 | 1 km, Daily |
|  | Emis_32 |  |
|  | LST_Night_1km |  |
|  | LST_Day_1km |  |
| CFSV2: NCEP Climate Forecast System Version 2, 6-Hourly Products | Maximum_specific_humidity_at_2m_height_above_ground_6_hour_interval | 22.26 km, 6-hour |
|  | Minimum_specific_humidity_at_2m_height_above_ground_6_hour_interval |  |
|  | Specific_humidity_height_above_ground |  |
|  | Maximum_temperature_height_above_ground_6_hour_interval |  |
|  | Minimum_temperature_height_above_ground_6_hour_interval |  |
|  | Geopotential_height_surface |  |
| MCD19A2.061: Terra & Aqua MAIAC Land Aerosol Optical Depth Daily 1km | Optical_Depth_047 | 1 km, Daily |
|  | Optical_Depth_055 |  |

**Meteorological variables:** Air pollution can be affected spatially and temporally by meteorological factors such as temperature, wind speed and direction, precipitation, humidity, and cloud droplets, the team collected satellite datasets from the 5^th^ generation European Center for Medium-Range Weather Forecasts atmospheric reanalysis (ERA5)^3^, which is archive at the Google Earth Engine. The ERA5 data (~9 to 11km^2^) includes average air temperature at 2m height (daily average), skin temperature (daily average), soil temperature in layer 1 (daily average), total precipitation (daily sums), surface pressure (daily average), 10m u-component of wind (daily average), and 10m v-component of wind (daily average).

Additionally, from the National Centers for Environmental Prediction (NCEP) Climate Forecast System (CFS), we collected maximum/minimum/mean specific humidity at 2m height (6-hour average), maximum/minimum temperature at 2m height (6-hour average) and geopotential height at surface (6-hour average). Also, from the Terra Moderate Resolution Imaging Spectroradiometer (MODIS), we used daytime/nighttime land surface temperature (daily average), emissivity with band 31 and 32 (daily average) and surface reflectance with band 1,2,3 and 7 (daily average).

**Aerosol variables:** The team used two aerosol optical depth variables: Aerosol optical depth over land retrieved in the MODIS Green band (0.55 μm) and aerosol optical depth over land retrieved in the MODIS blue band (0.47 μm). Aerosol optical depth is not received at high altitude (greater than 4.2 km) except when smoke or dust is detected; rather, this value reports a static value of 0.02 used for atmospheric correction.

**Vegetation variables:** The variables related to the vegetation in each grid were collected in the following two datasets in Google Earth Engine. With ERA5 dataset, we collected one-half of the total green leaf area per unit horizontal ground surface area for high vegetation type (daily average) and one-half of the total green leaf area per unit horizontal ground surface area for low vegetation type (daily average). Also, with MODIS dataset, we used the 16-day averaged Enhanced Vegetation Index (EVI), the second vegetation layer that can mitigate canopy background variations, preserve sensitivity in dense vegetation, and utilize the blue band to eliminate residual atmosphere contamination from smoke and sub-pixel thin cloud clouds.

**(2.2) Regional data**

**Population density:** We used population density data from the community health-related factor database sourced from the Korea Centers for Disease Control and Prevention, which comprises data pertaining to health determinants encompassing physical, environmental, and demographic factors influencing community health levels and disparities. Therefore, as population density data was collected at a Korean district-level (‘Si-gun-gu’) and its spatial resolution is bigger than 1km^2^, the same value was assigned to the grids which were included in each district.

**(3) Machine learning modeling and model ensemble**

Three machine learning-based models, namely random forest, light gradient boosting, and deep neural network, were used to predict daily PM_2.5_ and ozone averages (individually) with a 1 km^2^ grid during 2015–2022. The team trained algorithm individually on all input variables (daily satellite variables with EVI and population density variables) and parameters of each algorithm selected by cross-validated grid search. To avoid overfitting, we validated each model with a cross-validation. In addition, because the number of monitoring stations in Korea has increased every year during the study period, we did a year-stratified cross-validation: training with 80% of the data and testing a prediction performance at the remaining 20% of data by each study year (from 2015 to 2022). From these procedures, we found the optimized model for each algorithm and calculated 1km^2^ daily predicted PM_2.5_ and ozone concentrations.

Furthermore, in order to increase the prediction performance, we performed an ensemble approach using the generalized additive model (GAM) to incorporate these three algorithms.^3^ This GAM model allows to address a flexible regression for the monitoring concentrations against the estimates from each machine learning algorithm, and annual land-use variables by thin plate splines. In here, we considered annual land-use variables collected from MODIS through the Google Earth Engine (the Table below)

| **Data source** | **Predictor variables** | **Collection Period** | **Spatiotemporal**  **Resolution** |
| --- | --- | --- | --- |
| Copernicus Global Land Cover Layers: CGLS-LC100 Collection 3 | Bare-coverfraction | 2015~2019 | 100m |
|  | Crops-coverfraction |  |  |
|  | Grass-coverfraction |  |  |
|  | Shrub-coverfraction |  |  |
|  | Tree-coverfraction |  |  |
|  | Urban-coverfraction |  |  |
|  | Water-permanent-coverfraction |  |  |
|  | Water-seasonal-coverfraction |  |  |
|  | Forest_type |  |  |
| GlobCover: Global Land Cover Map | Landcover | 2009~2010 | 300m |
| MCD12Q1.061 MODIS Land Cover Type Yearly Global 500m | LC_Type1 | 2002~2021 | 500m, Yearly |
|  | LC_Type2 |  |  |
|  | LC_Type3 |  |  |
|  | LC_Type4 |  |  |
|  | LC_Type5 |  |  |
|  | LC_Prop1 |  |  |
|  | LC_Prop2 |  |  |
|  | LC_Prop3 |  |  |
|  | LC_Prop1_Assessment |  |  |
|  | LC_Prop2_Assessment |  |  |
|  | LC_Prop3_Assessment |  |  |
| MOD13A2.061 Terra Vegetation Indices 16-Day Global 1km | EVI | 2002~2020 | 1km, 16-day |

**Land-use variables:** To consider regional heterogeneity, we collected several land-use variables. From the Copernicus Global Land Service (CGLS), we used forest type with tree percentage vegetation cover bigger than 1% and percent vegetation cover for the various land cover classes including bare-sparse-vegetation, cropland, herbaceous vegetation, shrubland, forest, build-up, permanent water and seasonal water. Also, with the global land cover map based on ENVISAT's Medium Resolution Imaging Spectrometer (MERIS) Level 1B data, we applied the land cover map data.

Furthermore, there are the yearly land-use data provided by MODIS dataset. The data contains annual International Geosphere-Biosphere Programme (IGBP) classification, annual University of Maryland (UMD) classification, annual Leaf Area Index (LAI) classification, annual BIOME-Biogeochemical Cycles (BGC) classification, annual Plant Functional Types classification, LCCS1 land cover layer (confidence), LCCS1 land cover layer (confidence) and LCCS3 surface hydrology layer (confidence). As the data provides yearly data, we assigned the same value to the daily values which were included in each year.

**(3) Performance of the GAM Ensemble Prediction Model**

Among the three pollutant models with single algorithm and the GAM ensemble model, the GAM ensemble model showed the best prediction accuracy for both PM_2.5_ and ozone. Thus, we summarized the performance of the GAM ensemble model below:

**Figure.** Spatial distribution of the modeled ozone (ppm) and PM_2.5_ (μg/m^3^) from GAM ensemble prediction models (2015-2019 average values at 1km^2^ scale)

**
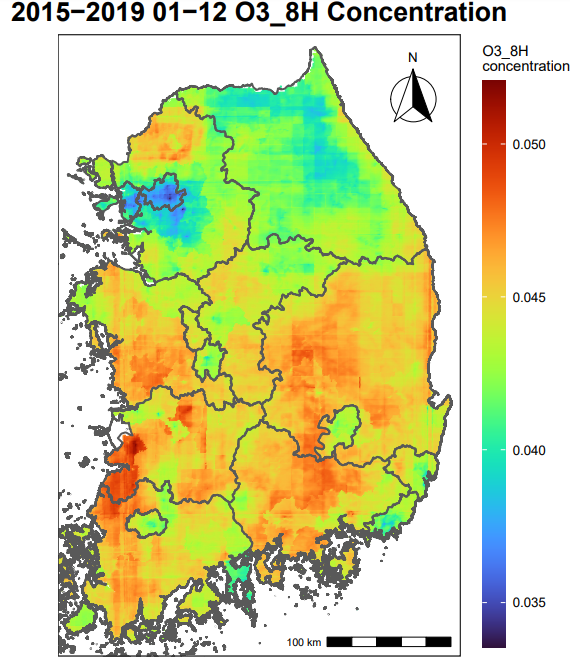
**
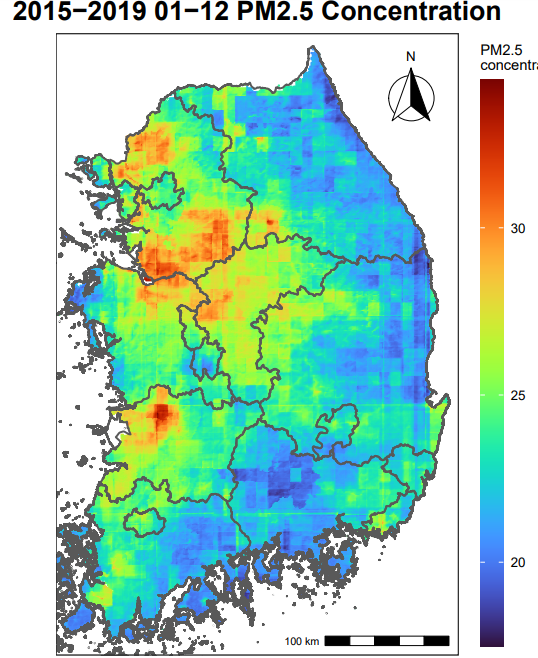


**2. Supplementary Tables**

**Table S1. Summary statistics on the performance of the PM_2.5_ and ozone prediction models during the study period (2015 to 2019).** RMSE: root mean squared error, MAE: mean absolute error. RMSE and MAE are in the unit PM_2.5_ of μg/m^3^ and ozone (ppm).

|  |  | **R^2^** | **RMSE** | **MAE** |
| --- | --- | --- | --- | --- |
| **PM_2.5_** | **Total years** | 0.944 | 3.219 | 2.187 |
|  | **2015** | 0.889 | 3.375 | 2.179 |
|  | **2016** | 0.882 | 3.352 | 2.290 |
|  | **2017** | 0.925 | 3.283 | 2.258 |
|  | **2018** | 0.957 | 3.453 | 2.148 |
|  | **2019** | 0.965 | 3.135 | 2.138 |
| **Ozone** | **Total years** | 0.944 | 0.004 | 0.002 |
|  | **2015** | 0.889 | 0.004 | 0.002 |
|  | **2016** | 0.882 | 0.004 | 0.002 |
|  | **2017** | 0.925 | 0.004 | 0.002 |
|  | **2018** | 0.957 | 0.004 | 0.002 |
|  | **2019** | 0.965 | 0.004 | 0.002 |

**Table S2. Distributions of absolute temperatures with their corresponding percentiles**

| **(%)** | **Temperature (ranges) (℃)** |
| --- | --- |
| **20** | 2.18 (-1.46, 7.13) |
| **70** | 19.79 (16.20, 20.79) |
| **71** | 20.01 (16.37, 21.02) |
| **72** | 20.24 (16.65, 21.24) |
| **73** | 20.47 (16.84, 21.44) |
| **74** | 20.69 (17.18, 21.61) |
| **75** | 20.92 (17.44, 21.85) |
| **76** | 21.13 (17.78, 22.07) |
| **77** | 21.36 (17.95, 22.32) |
| **78** | 21.58 (18.25, 22.57) |
| **79** | 21.80 (18.52, 22.84) |
| **80** | 22.02 (18.78, 23.04) |
| **81** | 22.23 (18.93, 23.31) |
| **82** | 22.44 (19.17, 23.48) |
| **83** | 22.66 (19.38, 23.65) |
| **84** | 22.90 (19.64, 23.90) |
| **85** | 23.16 (19.94, 24.10) |
| **86** | 23.42 (20.12, 24.40) |
| **87** | 23.70 (20.41, 24.68) |
| **88** | 24.01 (20.63, 24.96) |
| **89** | 24.32 (21.06, 25.30) |
| **90** | 24.64 (21.37, 25.67) |
| **91** | 24.98 (21.69, 25.96) |
| **92** | 25.32 (22.18, 26.24) |
| **93** | 25.70 (22.61, 26.64) |
| **94** | 26.10 (22.95, 27.12) |
| **95** | 26.52 (23.31, 27.63) |
| **96** | 26.97 (23.76, 28.14) |
| **97** | 27.46 (24.19, 28.90) |
| **98** | 27.93 (24.78, 29.41) |
| **99** | 28.64 (25.54, 30.19) |

**Table S3. Sensitivity analysis results.** Numbers: Lag-cumulative ORs between a reference temperature percentile (20^th^ percentile) and MaxST in the total population and by urbanicity level. OR: Odds ratio. MaxST: Maximum suicide temperature percentile.

|  | | **Total Population** | **Metropolitan areas** | **Mid-Urban**  **areas** | **Less-Urban areas** |
| --- | --- | --- | --- | --- | --- |
| **Main analysis** | | 1.28 (1.09,1.50) | 1.47 (1.14,1.90) | 1.03 (0.93,1.14) | 1.63 (0.97,2.75) |
| **Temperature, Lag days** | **Lag 0-3** | 1.29 (1.09,1.53) | 1.45 (1.13,1.87) | 1.06 (0.83,1.37) | 1.57 (0.92,2.68) |
|  | **Lag 0-5** | 1.35 (1.13,1.61) | 1.49 (1.14,1.95) | 1.12 (0.85,1.47) | 1.68 (0.99,2.86) |
| **Temperature, df** | **df=3** | 1.29 (1.10,1.51) | 1.48 (1.14,1.93) | 1.05 (0.90,1.23) | 1.72 (0.91,3.24) |
|  | **df=5** | 1.28 (1.09,1.50) | 1.52 (1.12,2.06) | 1.03 (0.91,1.16) | 1.60 (0.94,2.73) |
| **Cofounders** | **Without PM2.5** | 1.26 (1.08,1.48) | 1.45 (1.14,1.85) | 1.03 (0.93,1.14) | 1.46 (0.87,2.43) |
|  | **Without O3** | 1.24 (1.08,1.42) | 1.40 (1.13,1.73) | 1.03 (0.93,1.14) | 1.38 (0.92,2.08) |
|  | **Without Dewpoint temperature** | 1.20 (1.10,1.31) | 1.24 (1.10,1.40) | 1.17 (1.02,1.34) | 1.12 (0.87,1.45) |
|  | **Without Holiday** | 1.27 (1.08,1.50) | 1.46 (1.14,1.89) | 1.03 (0.93,1.13) | 1.63 (0.96,2.77) |

**Supplementary Figure**

**Figure S1.** **Lag-cumulative associations between ambient temperature(°C) and suicide in the total population and by subgroup.** OR: Odd ratio (a reference point: 20^th^ percentile of the temperature distribution). Dashed lines indicate the maximum suicide risk temperature percentiles (MaxSTs).


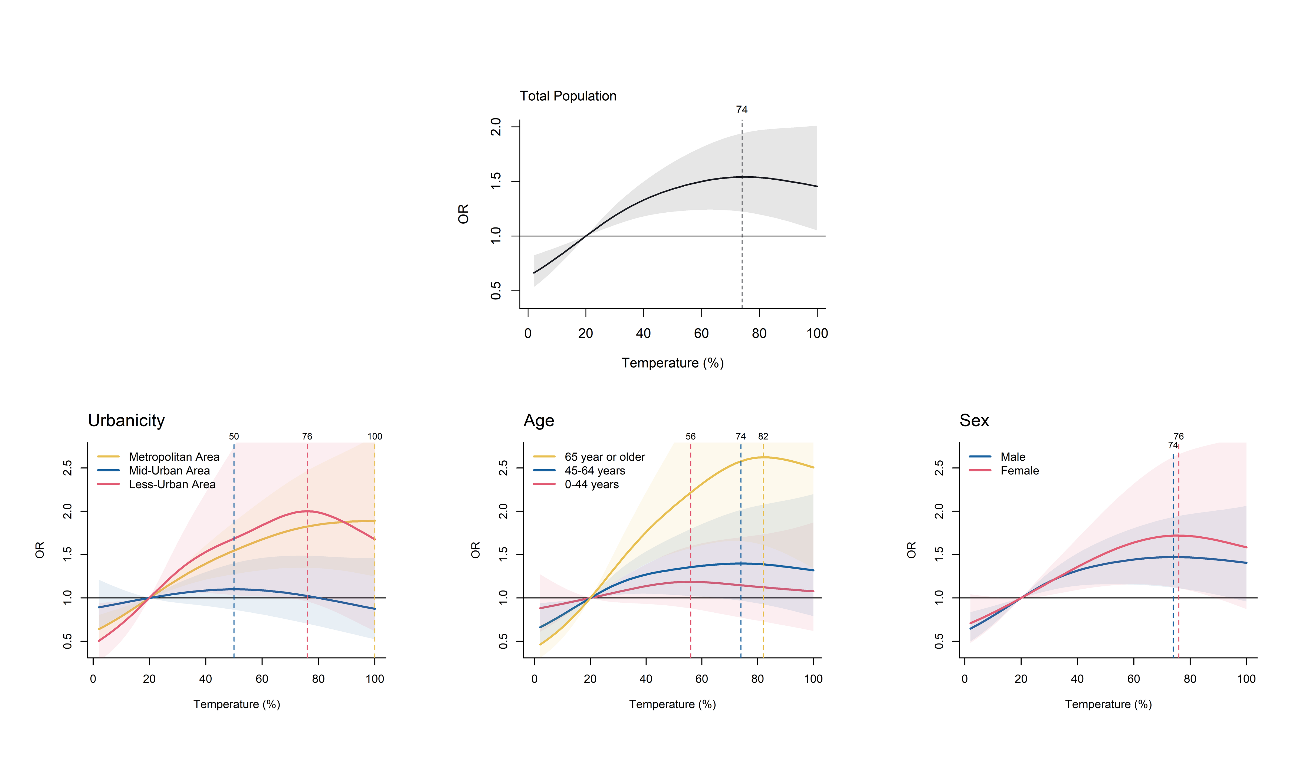


**Figure S2. Lag-cumulative ORs per a 10 °C increase in ambient temperatures in the total population and by urbanicity level and sex/age group.** A linear association between temperature and suicide was applied. OR: Odds ratio.


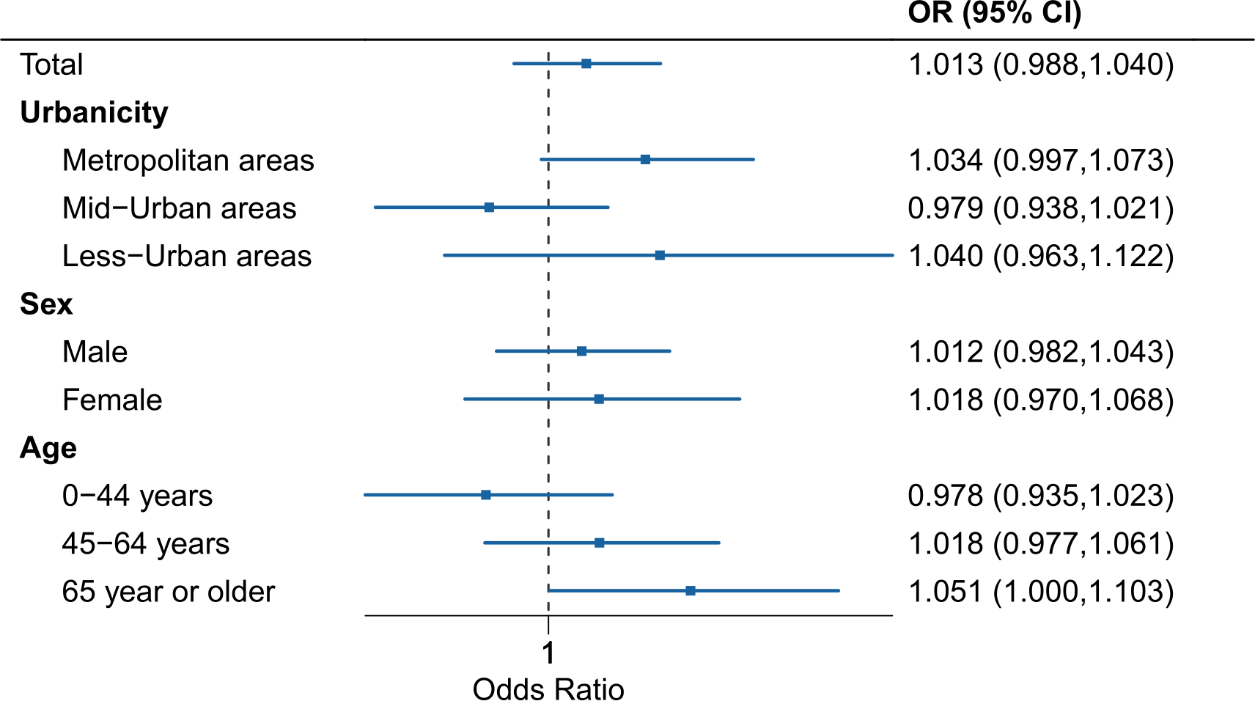


**Figure S3. Lag-cumulative ORs per a 10 °C increase in ambient temperatures by urbanicity level and sex/age groups.** A linear association between temperature and suicide was applied. OR: Odds ratio.


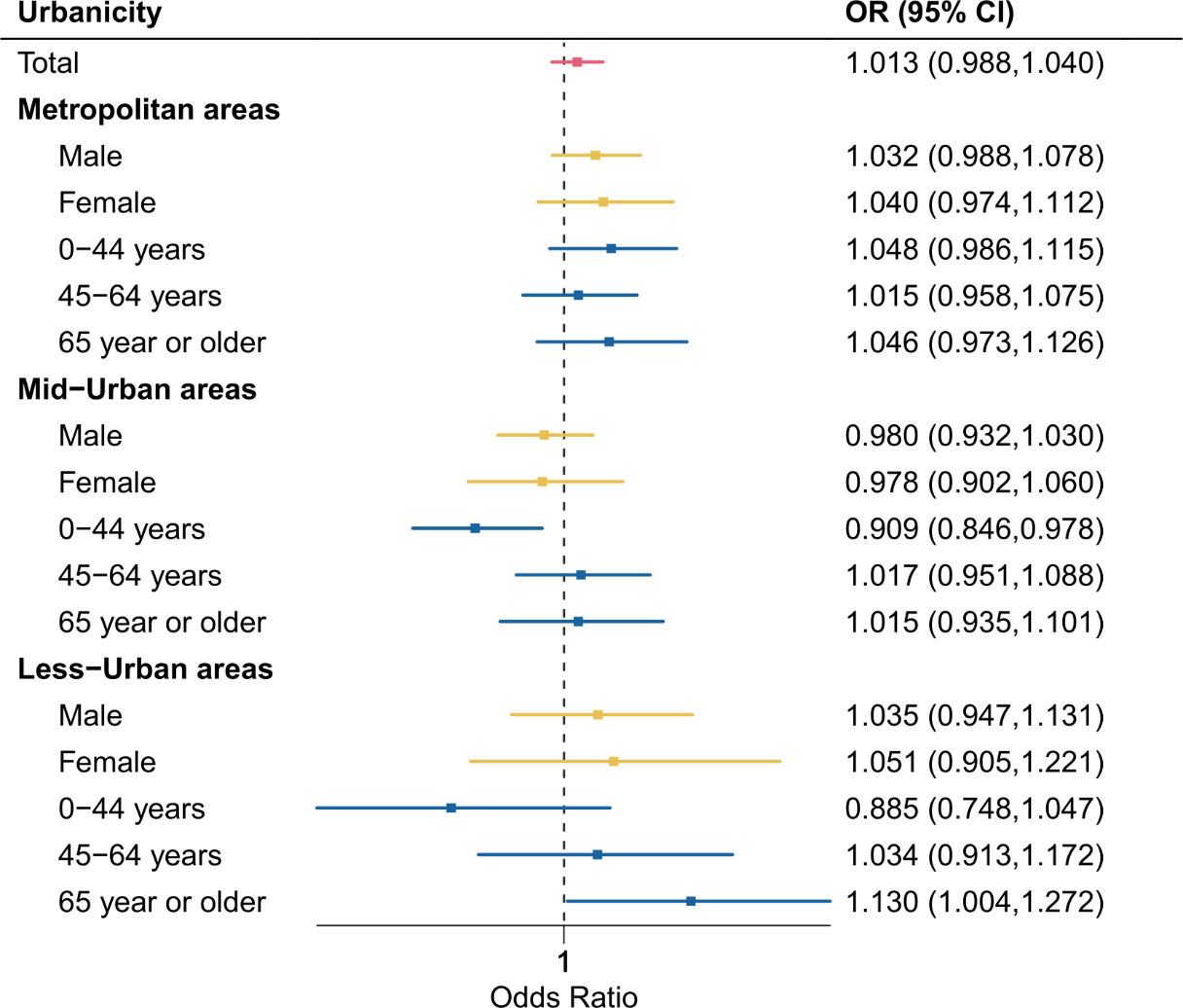


**Figure S4. Lag-cumulative ORs per a 10 °C increase in ambient temperatures by urbanicity-related indicators.** OR: Odds ratio. MaxST: Maximum suicide temperature percentile. The % of people who receive the National Basic Livelihood Security Service (% Basic livelihood security recipient), the % of people who could not visit the medical facilities when they wanted within a year (% Unmet medical needs), and the % of people who could not visit the medical facilities when they wanted within a year because of economic reasons (% Unmet medical needs due to economic reasons).


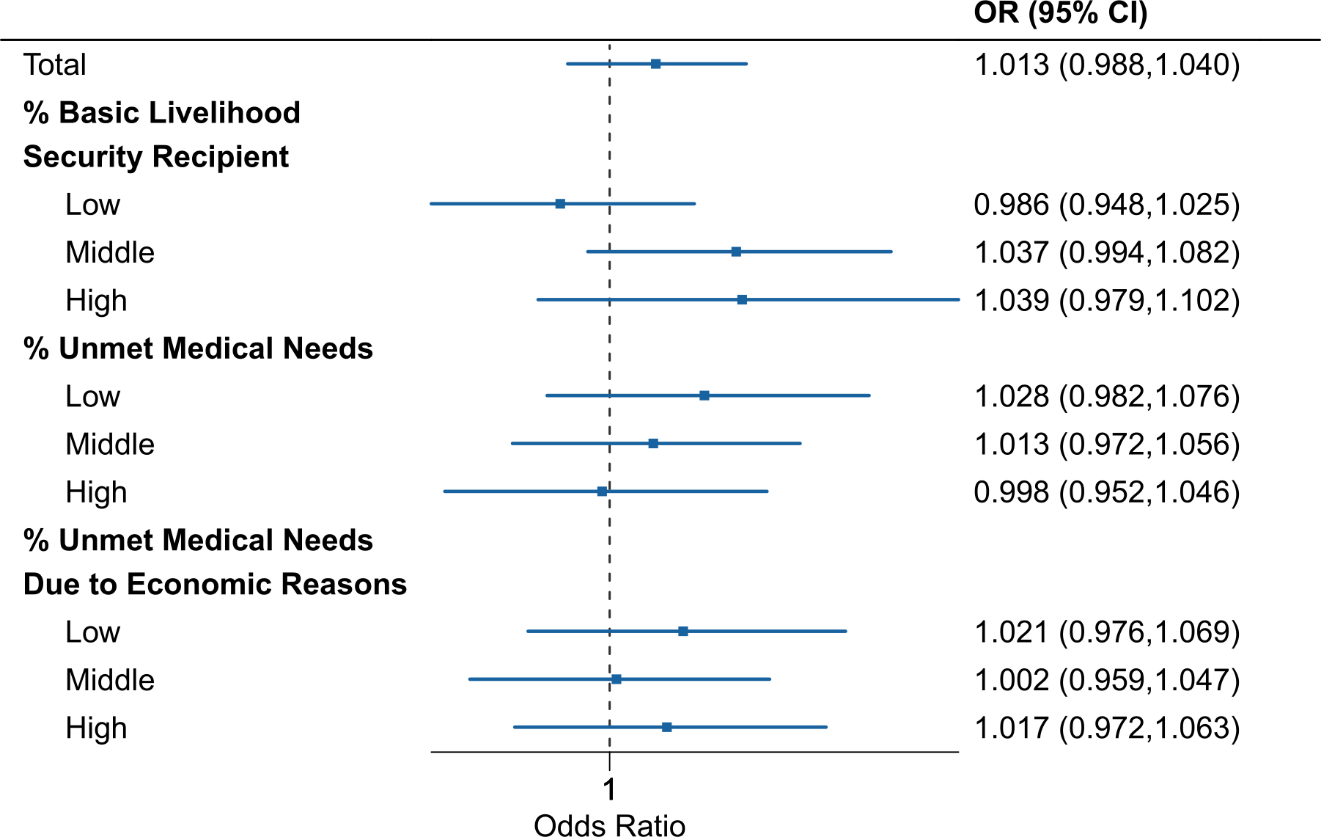


**Figure S5 (a)-(b). Sensitivity analysis results.** OR: Odd ratio (a reference point: 20^th^ percentile of the temperature distribution). Dashed lines indicate the maximum suicide risk temperature percentiles (MaxSTs).

(a) Lag-cumulative associations between ambient temperature and suicide in the total population and by subgroup (lag 0-3).


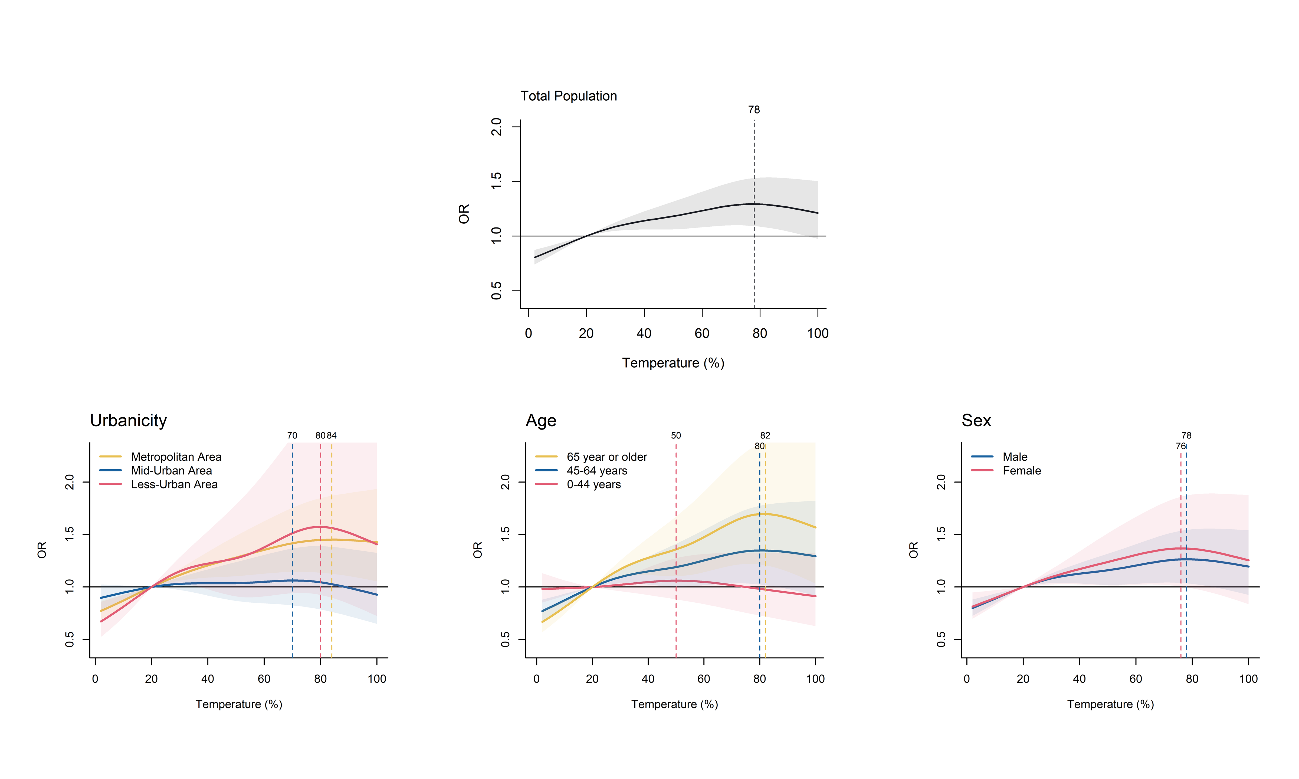


(b) Lag-cumulative associations between ambient temperature and suicide in the total population and by subgroup (lag 0-5).


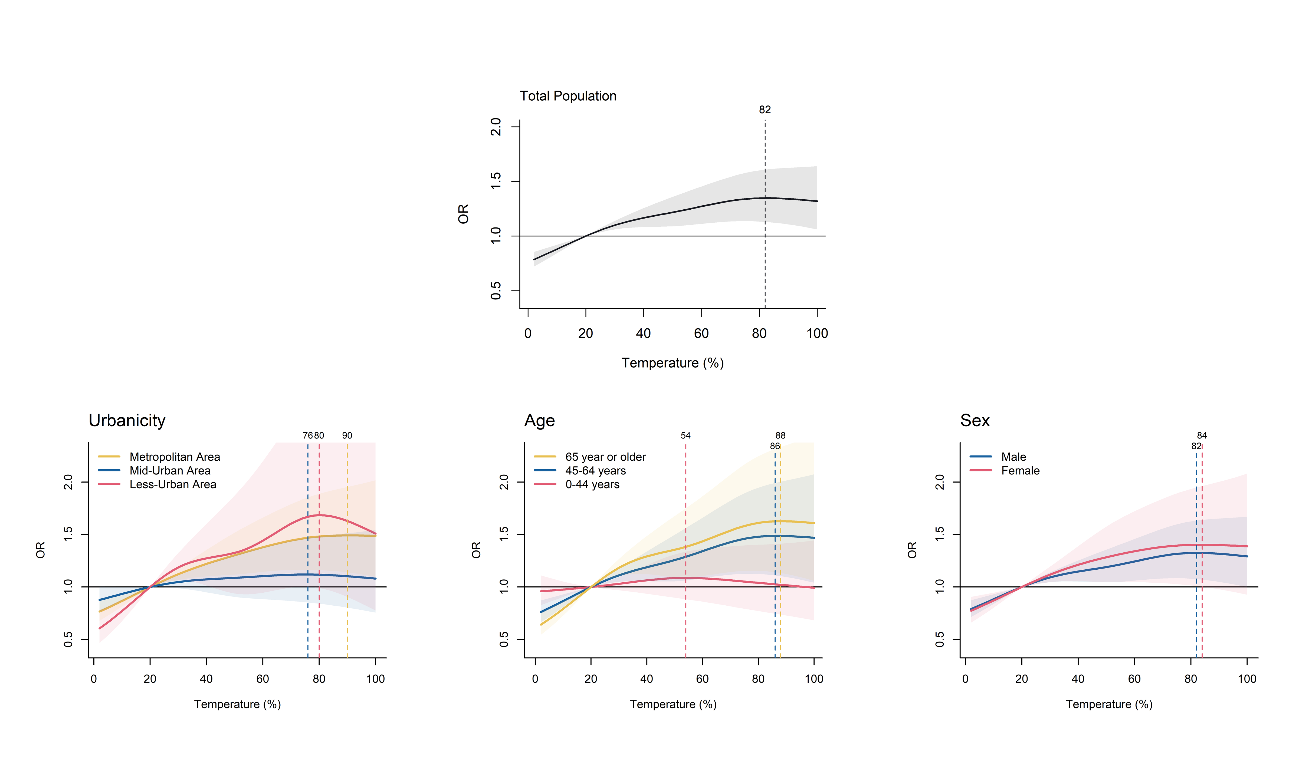


**References of the Supplementary Materials**

1. Jinah P, Cinoo K, Jieun M, et al. Association of long-term exposure to air pollution with chronic sleep deprivation in South Korea: A community-level longitudinal study, 2008–2018. *Environmental Research* 2023; **228**: 115812.

2. Kim H, Jang H, Lee W, et al. Association between long-term PM2.5 exposure and risk of Kawasaki disease in children: A nationwide longitudinal cohort study. *Environmental Research* 2023: 117823.

3. Di Q, Amini H, Shi L, et al. An ensemble-based model of PM2.5 concentration across the contiguous United States with high spatiotemporal resolution. *Environment International* 2019; **130**: 104909.
